# Circular RNAs: a new frontier for cancer diagnosis and therapy

**DOI:** 10.1186/s13045-018-0569-5

**Published:** 2018-02-13

**Authors:** Miaoci Zhang, Yan Xin

**Affiliations:** Laboratory of Gastrointestinal Onco-Pathology, Cancer Institute & General Surgery Institute, The First Affiliated Hospital of China Medical University, 155 Nanjing North Street, Heping District, Shenyang, Liaoning Province 110001 China

**Keywords:** CircRNA, Cancer, Biomarker, Therapy, Diagnosis, Prognosis

## Abstract

In recent years, circular RNAs (circRNAs) have attracted considerable attention because they play a significant role in many fields of cancer biology. Additionally, it has become increasingly clear that circRNAs have the potential to make contributions to the successful application of individualized cancer medicine. This brief review introduces circRNAs by describing their potential as a biomarker and therapeutic target and discussing the possible strategies to target them. This review also presents the challenges that are encountered by circRNAs for their definitive entry into clinical practice. Clearly, our understanding of circRNAs helps to add a new dimension to the molecular structure of cancer and will provide many new opportunities for cancer treatment.

## Background

In the last decade, the extensive application of new innovative technologies has promoted the fast development of many aspects of the RNA field [1]. In particular, next-generation sequencing has helped to realize the discovery of circular RNA (circRNAs) and bring circRNAs to the center stage in cancer genomics research [2, 3]. As a new type of RNA, circRNAs are expressed endogenously as covalently closed, single-stranded circular molecules [4]. Most circRNAs are the result of a “back-splice” reaction, during which an upstream acceptor splicing site is joined with a donor splicing site [5, 6]. Originating as exons, introns, or both, circRNAs fall into three categories [7]. Exonic circRNAs (ecRNAs) originate in the sequence of pre-mRNAs during the process of exon skipping. Reverse-oriented repeats that usually appear in the introns on both sides of the exons in ecRNAs can be important for the circularization of exons [8]. Intronic circRNAs (ciRNAs) that consist of one or more introns are derived from linear RNA. Specific 11-nt C-rich element near the branch-point site and 7-nt GU-rich motifs near the 5′ splice site are vital for the biosynthesis of ciRNA [9]. Exon-intron circRNAs (ElciRNAs) are another type of circRNA stemming from both introns and exons. The circularization of EIciRNAs can be improved by the flanking repeat sequence [10]. Among the three types of circRNAs, two are located in the nucleus (ciRNAs and EIciRNA) and one is cytoplasmic (ecRNA) [11]. Most genes with circular isoforms produce only one or two distinct circRNAs, although some produce tens of distinct circular products [12]. Although the mechanism of circRNA biogenesis cannot be understood completely, the complementary sequence in the flanking introns is the basis of the biogenesis for the circRNAs of many mammals [5, 13]. Additionally, some activating or antagonistic trans-acting factors, such as quaking and ADAR, can modulate the expression of mammalian circRNAs [14, 15]. Many scientific studies have demonstrated numerous potential functions of circRNAs that clarify how circRNA changes from negative matter to its current popular rank [16]. Most circRNAs are abundant and conserved across species and exhibit tissue-specific property [17]. Importantly, circRNAs are stably expressed in the saliva, blood, and exosomes, which makes them promising biomarkers for diagnosis, prognosis, and therapeutic response for cancer patients [18]. Furthermore, in recent years, several studies have indicated that circRNAs exhibited a powerful functional potential in regulating proliferation, apoptosis, angiogenesis, and metastasis, suggesting that circRNAs may be important target molecules in cancer [19, 20]. This review briefly introduces what is currently known about the potential of circRNA as a biomarker and therapeutic target in cancer and discusses their application prospects in the clinical field.

## Biological functions of circRNAs

### CircRNAs as microRNA and protein sponges

As a new type of competing endogenous RNA (ceRNA) that is widely believed to decoy relevant microRNAs (miRNAs), circRNAs can impact the bio-functions of miRNAs and thus play an important role in the regulation of miRNA target genes. Compared with other ceRNAs, circRNA has a far better ability to bind miRNAs; thus, it is also called a “super sponge”. This is best illustrated with the example of circular RNA sponge for miR-7 (ciRS-7), which sponges miR-7 efficiently with over 70 miR-7 binding sites [21, 22]. A high level of endogenous interactivity between them could be observed in the mouse brain, especially in the hippocampal and neocortical neurons [21]. Ectopic expression of human ciRS-7 mimicked the phenotype caused by knockdown of miR-7, indicating that ciRS-7 can act as a sponge [22]. Having a strong resistance to instability mediated by miRNA, it becomes a useful inhibitor of miR-7 activities, and the transcript levels of miR-7 targets are increased [21, 22]. Another RNA circle, originating at sex-determining region Y (SRY), is composed of 16 binding sites of miR-138, and it functions as the sponge of miR-138, thereby upregulating the expression of miR-138 target genes [21, 23].

Another new function of circRNA is that it can serve as a protein decoy which harbors binding sites for a single or multiple proteins and thus regulate gene expression. For example, both Argonaute (AGO) proteins and RNA polymerase II (Pol II) are correlated with circRNA [9, 24]. As “scaffolding” for RNA-binding proteins, circRNAs can facilitate steady interaction and bind multiple proteins because of their intrinsic stability. For instance, circFoxo3 binds to p21 and CDK2, leading to the formation of the ternary complex circFoxo3-p21-CDK2. Therefore, both the biological effect of CDK2 and the progression of the cell cycle can be arrested [25]. Another supported example is circMBL which sequesters MBL protein and prevents it from binding to other targets [5].

### CircRNAs as transcriptional regulators

Recent advances have revealed that circRNAs can play a transcriptional regulatory role. Located in the nucleus, ciRNAs in human cells can regulate the transcription of parent genes in cis. Certain ciRNAs, such as ci-sirt7, ci-mcm5, and ci-ankrd52, interact with the extended RNA Pol II complex. The transcription of sirt7, mcm5, and ankrd52 genes can be reduced via the depletion of ciRNAs, showing that the Pol II transcription of parent genes can be greatly enhanced through nuclear-enriched ciRNAs [9]. Additionally, accumulating evidence has revealed that EIciRNAs, such as EIciPAIP2 and EIciEIF3J, can interact with the U1 small nuclear ribonucleoprotein (snRNP) and RNA Pol II in the promoter region to realize the better transcription of the parent gene. Each EIciRNA contains one U1 small nuclear RNA (snRNA)-binding site at the retained introns. If the RNA-RNA interaction were blocked, the combination between Pol II and EIciRNA would be reduced, and fewer EIciRNA-U1 snRNP complexes would be bound to the coding gene promoter. Thus, the transcription of genes could be decreased [10].

### CircRNAs can be translated

Convincing evidence has shown that circRNAs can encode for proteins. When an internal ribosome entry site (IRES) is engineered in, circRNAs can be translated in vitro and in vivo effectively, showing that circRNAs can participate in translation [26, 27]. In addition, the translation of circRNA in the cells of human bodies can be realized merely through rolling circle amplification (RCA) without translational element [28]. Weingarten-Gabbay et al. conducted a survey revealing that massive circRNAs have a novel cap-independent translation mechanism [29]. Meanwhile, Legnini et al. and Pamudurti et al. also provided in vitro and in vivo evidence to prove that cap-independent translation exists in circRNAs [30, 31]. Additionally, Yang et al. found that human cells are pervasive with circRNA translation driven by *N*^6^-methyladenosine (m6A) [32].

## CircRNAs as potential biomarkers in cancer

CircRNAs are not sensitive to ribonucleases, such as exonuclease or RNase R, and have a longer half-life with a more prominent specificity in histology. Meanwhile, compared with detecting proteins through antigen-antibody reactions, testing circRNAs through quantitative real-time PCR (qRT-PCR) and in situ hybridization are more specific and sensitive. Herein, circRNAs are potential biomarkers in cancer [33].

### CircRNAs in cancer diagnosis

It is well known that most cancer types can be cured, if diagnosed at an early stage. Commonly used cancer diagnostic markers and tools include magnetic resonance imaging, computerized tomographic scanning, and histopathology. However, some of these methods are invasive and expensive. Therefore, minimally invasive and inexpensive methods are required. The fact that circRNAs are highly dysregulated in several cancer types and exhibit a high degree of tissue- and disease-specificity make them an ideal candidate for cancer diagnosis (Table 1). For example, hsa_circ_0013958 was demonstrated to be significantly upregulated in the tissue and plasma of lung adenocarcinoma (LAC). Furthermore, its levels were correlated with lymphatic metastasis and tumor-node-metastasis (TNM) stage and the area under the receiver operating characteristic (ROC) curve was 0.815, suggesting that it could be a promising biomarker for LAC [34]. In another study, Li et al. found that 257 new circRNA species were detected and 67 circRNAs were missing in colorectal cancer (CRC) patients compared with those in healthy controls based on serum exosome RNA sequencing (RNA-seq) datasets. Furthermore, it was confirmed that upregulation of serum circ-KLDHC10 was able to distinguish CRC patients from healthy subjects, suggesting that it is a promising non-invasive biomarker for the early detection and screening of CRC [35].

**Table 1:** A list of circRNAs with potential in cancer diagnosis

Cancer type	CircRNA	Expression	Source	Reference
Bladder cancer	CircMYLK	Up	Tissue	[52]
	CircTCF25	Up	Tissue	[85]
Prostate cancer	CircSMARCA5	Up	Tissue	[86]
Glioma	CircTTBK2	Up	Tissue	[87]
Osteosarcoma	Circ_0001564	Up	Tissue	[88]
Breast cancer	Circ_0001982	Up	Tissue	[89]
	Circ_103110, circ_104689 and circ_104821	Up	Tissue	[90]
	CircABCB10	Up	Tissue	[91]
Hepatocellular cancer	CiRS-7	Up	Tissue	[92]
	CircZFR	Up	Tissue	[93]
	CircFUT8	Up	Tissue	[93]
	CircIPO11	Up	Tissue	[93]
	Circ_0005075	Up	Tissue	[94]
Esophageal cancer	Circ_0067934	Up	Tissue	[95]
Colorectal cancer	CircBANP	Up	Tissue	[96]
	Circ_0020397	Up	Tissue	[97]
	CircKLDHC10	Up	Serum	[35]
Lung adenocarcinoma	Circ_0013958	Up	Serum	[34]

CircRNAs can also help in risk assessment of cancer susceptibility. Single nucleotide polymorphism (SNP) refers to a difference in a single DNA building block of a gene (nucleotide) or within the regulatory regions of a gene. SNPs can be used as a biomarker for predicting an individual’s risk of cancer [36]. A study from China consisting of 1600 ethnic Han patients with hepatocellular cancer (HCC) and 1800 cancer-free controls, six tagSNPs were identified in circITCH: rs6059851, rs6120663, rs10485505, rs4911154, rs7266300, and rs11167234. Consequently, rs10485505 and rs4911154 were significantly associated with the increased risk of HCC [37]. This study clearly indicates an association of circRNA SNPs with cancer susceptibility. However, how circRNA SNPs contribute to cancer pathogenesis is not properly defined. The larger, well-designed in-depth studies from different ethnic populations will validate contributions of circRNA SNPs in cancer risk assessment.

The above examples are only a fraction of the several instances in which circRNAs have demonstrated promising diagnostic potential. More studies are required before circRNAs can be recommended for human use.

### CircRNAs in cancer prognosis

Prognostic evaluation is of great significance for the early intervention of poor prognostic factors and the prolongation of the life expectancy of cancer patients. Recently, a great deal of research showed that examination of circRNA may be useful in the prognostic prediction of cancer (Table 2). For instance, glioblastoma patients with higher circ-FBXW7 had an increased overall survival (OS) compared with those with low expression [38]. In addition, it was found that upregulated circUBAP2 in osteosarcoma tissues was significantly associated with a lower survival rate [39]. Furthermore, circ_100876 upregulation was closely related to shorter OS, lymphatic metastasis and tumor staging in non-small cell lung cancer patients [40].

**Table 2:** A list of circRNAs with potential in cancer prognosis

Cancer type	CircRNA	Expression	Source	Association	Reference
Glioblastoma	CircFBXW7	Down	Tissue	OS	[38]
Osteosarcoma	CircUBAP2	Up	Tissue	TNM and OS	[39]
Lung cancer	Circ_100876	Up	Tissue	Lymph node metastasis,TNM and OS	[40]
	CircITCH	Down	Tissue	TNM	[53]
Laryngeal cancer	Circ_100855	Up	Tissue	Lymph node metastasis and TNM	[98]
	Circ_104912	Down	Tissue	Lymph node metastasis and TNM	[98]
Bladder cancer	CircHIPK3	Down	Tissue	Vascular invasion and lymph node metastasis	[99]
Colorectal cancer	CircCCDC66	Up	Tissue	OS	[59]
	Circ_001569	Up	Tissue	Distant metastasis, differentiation, and TNM	[100]
	Circ_103809	Down	Tissue	TNM	[101]
	Circ_104700	Down	Tissue	Distal metastasis	[101]
	CiRS-7	Up	Tissue	Lymph node, distant metastasis, and OS	[51]
Hepatocellular cancer	Circ_0003570	Down	Tissue	Metastasis	[102]
	CircMTO1	Down	Tissue	OS	[103]
	Circ_100338	Up	Tissue	Cumulative survival rate, lung metastasis, vascular invasion, and TNM	[104]
	CircZKSCAN1	Down	Tissue	Microscopic vascular invasion and TNM	[60]
Gastric cancer	Circ_104916	Down	Tissue	TNM and lymph node metastasis	[105]
	Circ_0006633	Down	Tissue	Distal metastasis	[106]
	Circ_0000096	Down	Tissue	TNM	[61]
	Circ_0000181	Down	Tissues and plasma	Lymphatic metastasis and distal metastasis	[107]
	Circ_100269	Down	Tissue	OS	[108]
	Circ_002059	Down	Tissues and plasma	TNM and metastasis	[109]
	Circ_0000190	Down	Tissue	TNM, lymphatic metastasis, and distal metastasis	[110]
	CiRS-7	Up	Tissue	OS	[50]
	Circ_0014717	Down	Tissue	Distal metastasis and TNM	[111]
	Circ_0003159	Down	Tissue	Distal metastasis and TNM	[112]

*Abbreviations*: *OS* overall survival, *TNM* tumor-node-metastasis

The currently available cancer drugs cannot eradicate cancer cells completely, and most cancer types frequently relapse over a period of time. Therefore, biomarkers that can predict tumor recurrence could be of prognostic significance. Recently, several studies showed that circRNAs could be utilized as potential biomarkers for the prediction of tumor recurrence. For example, the expression of hsa_ circ_0058246 was statistically significantly increased in patients who suffered from the recurrence of gastric cancer (GC) [41]. In another study, patients with lower levels of circPVT1 in GC had a significantly shorter progression-free survival (PFS) than those with high levels of circPVT1, indicating that circPVT1 might be a biomarker for tumor recurrence [42]. Additionally, circBRAF could also be a candidate biomarker for predicting glioma recurrence [43]. Hence, circRNAs have shown potential as biomarkers for predicting recurrence in several cancer types, which could help the clinicians to manage disease before recurrence.

In conclusion, both upregulation and downregulation of circRNA expression could be of prognostic significance. We believe that the use of multiple sensitive and reliable detection methods in large-scale, multi-center studies will help to unravel the true prognostic significance of circRNAs in cancer patients.

### CircRNAs in predicting response to chemoradiotherapy

Recent studies have found that circRNAs impact the sensitivity of tumors to chemoradiotherapy. For instance, Su et al. revealed that circ_001059, circ_100385 and circ_104983 had the largest upregulation; circ_000695, circ_101877, and circ_102913 had the largest downregulation in radioresistant oesophageal cancer cells according to the microarray data, indicating that these dysregulated circRNAs may be involved in the development of radiation resistance [44]. Another recent study showed that certain fusion-circRNAs suggest resistance to apoptosis-inducing chemotherapy, such as f-circM9, which confers protection to leukemic cells upon treatment with arsenic trioxide [45]. Additionally, Xiong et al. performed microarray analysis of 5-fluorouracil (5-FU) chemoradiation-resistant CRC cells and identified hsa_circ_0007031, hsa_circ_0000504, and hsa_circ_0007006 that are the top three upregulated circRNAs among differentially expressed circRNAs, indicating that they are potential biomarkers to predict chemoradiation resistance in CRC [46]. A study conducted by Gao et al. revealed that the expression of hsa_circ_00006528 in adriamycin (ADM)-resistant human breast cancer tissues and cell lines were obviously higher than that in ADM-sensitive groups, suggesting the potential of circ_0006528 in predicting chemotherapy resistance in breast cancer [47].

In conclusion, circRNAs as biomarkers predicting chemoradiotherapy response in individual patients are essential for improving the clinical management of cancer.

## Therapeutic potential of circRNAs in cancer

### CircRNA as potential therapeutic targets

An increasing amount of evidence has demonstrated the relationship between various novel circRNAs and signaling pathways with carcinogenesis or with the manipulation of aggressive characteristics of cancer cells, both of which are putative therapeutic targets for novel drugs to increase the survival of cancer patients [48, 49]. One example is ciRS-7, which was significantly upregulated in GC tissues compared with matched para-carcinoma specimens. Further consequences revealed that ciRS-7 promoted a more aggressive oncogenic phenotype by blocking the miR-7-mediated PTEN/PI3K/AKT signaling pathway [50]. Weng et al. revealed that ciRS-7 was significantly elevated in CRC. Significantly higher ciRS-7 levels were found to be related to lymph node involvement, more advanced II to IV stages and distant metastasis. In addition, ciRS-7 is an independent risk factor for OS. Mechanistically, ciRS-7 can regulate the EGFR/RAF1/MAPK signaling pathway by competing for miR-7 in CRC [51]. In another study, circRNA-MYLK was significantly upregulated in bladder cancer (BC). Importantly, circRNA-MYLK levels were related to the progression of the stage and grade of BC. Mechanistically, circRNA-MYLK might function as ceRNA for miR-29a, which could contribute to the epithelial-mesenchymal transition and the development of BC through activating VEGFA/VEGFR2 and the downstream Ras/ERK signaling pathway. These data suggest that circRNA-MYLK would be a promising target for BC therapy [52]. Another example is circITCH, which positively regulates ITCH by sponging miR-7. ITCH can inhibit the Wnt/β-catenin pathway and thus circITCH can suppress lung cancer, oesophageal squamous cancer and CRC from progression partially via inhibiting the Wnt/β-catenin pathway [53–55]. Therefore, circularization may be the future target of cancer treatment.

### CircRNAs as potential therapeutic vectors

The superior ability of circRNA in sponging proteins and miRNAs and its unique cellular stability make it a promising therapeutic vector. Cancer may be stopped by restoring the normal regulatory network through exogenous introduction of circRNA which has multiple binding sites for oncogenic proteins and/or miRNAs [56, 57]. The sponging repertoire can include combinations of miRNA and protein binding sites specifically designed to target specific oncogenic profiles. Furthermore, as the expression of circRNA is regulated by Pol II promoters, we can use disease-activated control elements to limit circRNA expression to malignant cells or use cell-specific promoters to control circRNA expression to certain cell types [19]. Herein, circRNA is a promising vector for cancer therapy.

## Therapeutic strategies targeting circRNAs

### Antagonizing circRNA function

#### Antagonizing circRNAs by short interfering RNAs

**Fig. 1:**
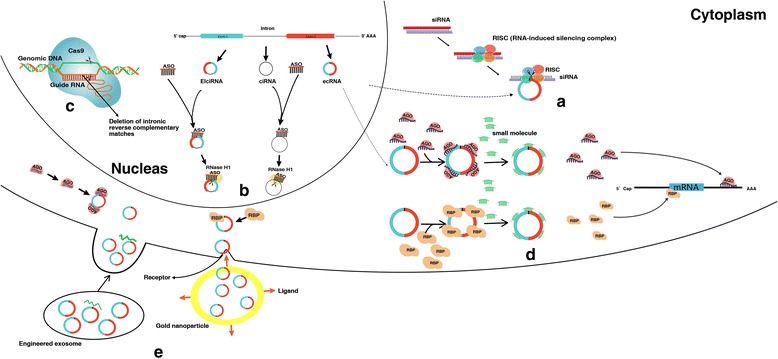
Possible approaches to target circRNAs. **a** siRNA-mediated circRNA-specific silencing. **b** ASO-mediated circRNA-specific silencing. **c** knockdown or knockout circRNA using CRISPR/Cas9. **d** Blockade of the combination between circRNA and miRNA or RBP by saturating the binding sites conserved on circRNA. **e** Reintroduction of circRNAs that are miRNA or RBP sponges

A viable approach to silence circRNA might be to target its unique back-splice junction by exogenously delivering a short interfering RNA (siRNA) perfectly complementary to this site (Fig. 1a). It is important to note that using siRNA to antagonize circRNA should be carried out in a manner that does not interfere with linear mRNA expression [58]. In recent years, siRNA has been applied to effectively silence circRNA in in vivo and in vitro studies [59–61]. However, the off-target effect is a most noteworthy problem that needs to be carefully avoided [62].

#### Antagonizing circRNAs by anti-sense oligonucleotides

Anti-sense oligonucleotides (ASOs), which are short-stranded single sequences, are used as an alternative to target the primary structure of circRNAs (Fig. 1b). It is feasible to knockdown ElciRNA or ciRNA using ASOs targeting intron sequences or back-splicing junction. For instances, Zhang et al. can efficiently knock down ciRNA with appropriate ASO targeting intron sequence [9]. Meanwhile, Li et al. successfully designed ASOs complementary to intron sequences or to the back-splicing junction in the EIciRNA [10]. A possible alternative approach to silence circRNA by exogenous ASOs is to interfere with the back-splicing signals in the pre-mRNA, such as the binding sites for trans-acting splicing factors or the flanking intronic Alu repeats [19]. Additionally, ASOs should be designed against circRNAs carefully. Otherwise, off-target effects of cognate parent gene suppression might be incurred [63].

#### Antagonizing circRNAs by CRISPER/Cas system

Clustered regularly interspaced short palindromic repeats-associated nuclease Cas9 (CRISPR/Cas) can function quickly and efficiently to cause circRNA to be partially or totally deleted without impeding the linear mRNA based on the deletion of one pair of a flanking intronic reverse complementary match (Fig. 1c). For example, in vitro, when the intronic RNA pairing across the circle-forming exons was disrupted by CRISPR-Cas9-mediated genome editing at the examined circRNA-producing locus, circGCN1L1 could not be detected at this locus at all [64]. Another successful example was circHIPK3, which was demonstrated by Zheng et al. [65]. Additionally, Rajewsky et al. successfully created an in vivo loss-of-function model for Cdr1as from the mouse genome using CRISPR/Cas9 with Cas9 mRNA and two sgRNAs designed to bind upstream of Cdr1as splice sites [66]. Herein, CRISPR/Cas9 technology offers an unprecedented opportunity for partial or complete removal of oncogenic circRNA.

#### Antagonizing circRNAs by blocking molecular interactions

When the molecular interactions of circRNAs are blocked, they will be impaired. When small-molecule inhibitors shield the binding points in the partners of protein interaction binding to circRNAs, the above description can be realized. There is another basic principle of the method, that is, to block the combination between circRNA and miRNA by making the conserved binding sites on circRNA saturated (Fig. 1d). Additionally, this is expected to provide a new viewpoint for cancer therapy [67, 68].

### Restoring circRNA function

When circRNA acts as a sponge for RNA-binding proteins (RBPs) or miRNAs, reintroduction of circRNA may restore controlled proliferation of the cancer cells or induce apoptosis (Fig. 1e). To date, several studies take advantage of a minigene construct containing only the exon to be circularized and parts of the flanking introns to express the circRNA of interest [5, 21, 69, 70]. However, although often used as a very convenient tool that allows overexpression of circRNAs in vitro, additional linear and circRNA products are often produced [69, 70]. Hence, to further research circRNAs, minigene construct design should be improved and alternative methods should be developed to facilitate circRNA overexpression [71].

## Delivery of circRNAs

Extracellular vesicles (EVs) are small lipid bilayer vesicles released by cells [72], which can be retooled as delivery vectors for therapeutic intervention [73, 74]. Recently, circRNAs were found to be both enriched and stable in the EVs of cancer cells [35, 75, 76]. In addition, circRNAs in cancer cells can be delivered to exosomes [77]. Furthermore, cells may use EVs to transport circRNAs to communicate to other cells [4]. Therefore, it is possible that EVs, including exosomes and microvesicles, could be engineered to deliver circRNAs efficiently to a target tissue [48, 75].

Nanoparticles have emerged as another promising approach for the delivery of circRNAs. Wu et al. has shown that, in vivo, the circular RNA circ-Foxo3 can be delivered to inhibit tumor progression by conjugating the expression plasmids of circ-Foxo3 with gold nanoparticles [78]. Since nanoparticles are not able to enter the nucleus, the therapeutic approach can only focus on exonic circRNAs, which are mainly detected in the cytoplasm.

## Conclusions

The powerful functions and unique properties of circRNAs have made them the focus of scientific and clinical research. In this review, we summarize the recent findings on circRNAs and highlight their clinical value in cancer. First, circRNAs could act as promising biomarkers in cancer diagnosis, prognosis, recurrence, risk evaluation, and response to chemoradiotherapy. Second, circRNAs are potential therapeutic targets and promising vehicles for the delivery of therapeutics. Engineered circRNAs might be effective for sequestering oncogenic miRNAs and RBPs. Third, extracellular vesicles and nanoparticles have emerged as a promising approach for the delivery of circRNAs. In conclusion, circRNAs provide a new perspective for the diagnosis and treatment of cancer.

## Perspective

CircRNAs provide new insights into the “dark matter” of the human genome [79]. However, the field of circRNAs remains largely unexplored and much progress has to be made to incorporate circRNAs into clinical practice.

First, more controlled clinical studies comprising a large number of patients are required before cancer-specific circRNAs can be recommended for human use.

Second, more detailed studies on how to deliver circRNAs efficiently to the accurate action site with a long-term sustained effect and without immunological rejection are needed.

Third, the precise mechanisms of circRNA underlying the initiation and progression of cancer are worth an in-depth and extensive study, which is of significance for individual therapeutic regimens. Here are some recommendations for future research of circRNAs in cancer.

For the discovery of novel circRNA species involved in cancer, numerous algorithms have been used to detect genome-wide circRNA expression from RNA-seq data in the past few years, but there is little overlap in their predictions [12]. Herein, it is advisable to run more than one circRNA prediction algorithm to minimize the number of false positives in the absence of a gold standard algorithm.

For the stringent circRNA validation method, northern blotting is a more accurate way to fully characterize RNA species than qRT-PCR as no reverse transcription and amplification steps are part of the protocol [80]. Fluorescence in situ hybridization (FISH) provides spatial information about specific circRNAs, which is likely to be employed more in future circRNA cancer research [81].

To discover the detail binding sites for circRNA-miRNA and circRNA-RBP interactions, AGO2 RNA immunoprecipitation (RIP) combined with luciferase reporter assay, RIP followed by sequencing, and RNA pull down combined with mass spectrometry are suggested methods [82, 83].

To elucidate circRNA cellular roles, it is indispensable that exogenous synthetic molecules with engineered sequences should be designed to mimic or antagonize circRNAs. Furthermore, these molecules should be able to decoy or release miRNAs and RBPs. This research method is important to obtain circRNAs’ accurate epigenetic roles and corresponding mechanisms in cancer.

Dynamic analysis of aberrantly expressed circRNAs in cancer, such as in different stages or different sensitivities to a specific chemotherapy treatment, is very crucial for further understanding and clarifying the mechanism underlying cancer development and presenting potential therapeutic targets. A preliminary study found a large number of differentially expressed circRNAs between primary ovarian tumor and metastases using RNA-seq, which could offer a more robust classifier, for tumor subtypes or provide biomarkers for tumor screening and prognosis [84]. Another preliminary study on chemoresistant breast cancer provided several appealing targets for further functional analysis according to circRNA microarray data [47]. More comprehensive studies on those differentially expressed circRNAs will certainly gain more insights of the expression dynamics and functions of circRNAs in cancer.

We hope that one day, the appropriate and precise use of circRNAs in clinical diagnosis and treatment will become a new foothold for translational and precision medicine.

## Abbreviations

5-FU: 5-Fluorouracil
ADM: Adriamycin
AGO: Argonaute
ASO: Anti-sense oligonucleotide
BC: Bladder cancer
ceRNA: Competing endogenous RNA
circRNAs: Circular RNAs
ciRNA: Intronic circRNA
ciRS-7: Circular RNA sponge for miR-7
CRC: Colorectal cancer
CRISPR/Cas9: Clustered regularly interspaced short palindromic repeats-associated nuclease Cas9
ecRNA: Exonic circRNA
ElciRNA: Exon-intron circRNA
EV: Extracellular vesicle
FISH: Fluorescence in situ hybridization
GC: Gastric cancer
HCC: Hepatocellular cancer
IRES: Internal ribosome entry site
LAC: Lung adenocarcinoma
m6A: *N*^6^-Methyladenosine
miRNA: MicroRNA
OS: Overall survival
PFS: Progression-free survival
Pol II: RNA polymerase II
qRT-PCR: Quantitative real-time PCR
RBP: RNA-binding protein
RCA: Rolling circle amplification
RIP: RNA immunoprecipitation
RNA-seq: RNA sequencing
ROC: Receiver operating characteristic
SiRNA: Short interfering RNA
SNP: Single nucleotide polymorphism
snRNA: U1 small nuclear RNA
snRNP: Small nuclear ribonucleoprotein
SRY: Sex-determining region Y
TNM: Tumor-node-metastasis
